# Anthracycline-Free FLA-VEN with or Without Midostaurin in Newly Diagnosed Intermediate- and Adverse-Risk AML: A Single-Center Retrospective Study

**DOI:** 10.3390/cancers18193132

**Published:** 2026-09-27

**Authors:** Sinem Namdaroglu, Merve Kakcı, Berrak Şenol, Osman Can Öztürk, Ömer Şeker, Mehmet Can Uğur, Nanişe Gizem Fener, Semih Başcı, Hikmetullah Batgi

**Affiliations:** 1 Department of Hematology, Faculty of Medicine, Dokuz Eylül University, 35340 İzmir, Türkiye; 2 Department of Hematology, University of Health Sciences Türkiye, İzmir Tepecik Training and Research Hospital, 35020 İzmir, Türkiye

**Keywords:** acute myeloid leukemia, venetoclax, fludarabine, cytarabine, midostaurin, FLT3, anthracycline-free induction, real-world study

## Abstract

Background: Anthracycline-containing intensive chemotherapy remains a standard frontline approach for medically fit patients with acute myeloid leukemia (AML). We retrospectively evaluated an exploratory anthracycline-sparing regimen combining fludarabine, cytarabine, and venetoclax (FLA-VEN), with midostaurin added in FLT3-mutated cases, in selected patients with intermediate- or adverse-risk AML. Methods: This retrospective single-center study included 24 consecutive adults with newly diagnosed intermediate- or adverse-risk AML treated between March 2024 and March 2026. Patients with favorable-risk AML were excluded. The primary endpoints were complete remission (CR) and safety. Secondary endpoints included hematologic recovery, allogeneic hematopoietic stem cell transplantation (allo-HSCT) rate, relapse incidence, transplant-related mortality (TRM), and overall survival (OS). Results: The median age was 49 years (range, 22–64), and 16.7% of patients were aged ≥60 years. FMS-like tyrosine kinase 3 internal tandem duplication (FLT3-ITD) mutations were present in 8 patients (33.3%). Following one induction cycle, 21 of 24 patients (87.5%) achieved CR. Among FLT3-ITD-mutated patients, 7 of 8 (87.5%) achieved CR, and FLT3-ITD became undetectable by the available polymerase chain reaction (PCR) fragment assay in all seven responders before transplantation. The 60-day mortality rate was 0/24 (0%). Median neutrophil and platelet recovery times were 17 and 21 days, respectively. Twenty-one patients (87.5%) underwent allo-HSCT in first remission. With a database cutoff of 30 August 2026, Kaplan–Meier estimated OS was 83.3% (95% CI, 61.5–93.4%) at 12 months. The longer-term 24-month OS estimate was 54.4% (95% CI, 25.5–76.3%); however, this estimate was imprecise because only five patients remained at risk at 24 months. Median OS was not reached. Conclusions: In this small exploratory cohort, anthracycline-free and G-CSF-free FLA-VEN, with or without midostaurin, was associated with high remission rates, no early mortality, and frequent bridging to allo-HSCT. These findings support prospective controlled evaluation of this anthracycline-sparing strategy.

## Simple Summary

Intensive treatment for acute myeloid leukemia commonly includes an anthracycline drug and, in some regimens, granulocyte colony-stimulating factor. We reviewed 24 adults with newly diagnosed intermediate- or adverse-risk disease who received fludarabine, cytarabine, and venetoclax without either component; patients with FLT3-ITD also received midostaurin. After one induction cycle, 21 patients achieved complete remission, no patient died within 60 days, and all responders proceeded to allogeneic stem cell transplantation in first remission. Updated survival analysis showed estimated overall survival of 83.3% at 12 months and 54.4% at 24 months. These findings support prospective evaluation of this anthracycline-sparing strategy in controlled studies.

## 1. Introduction

Acute myeloid leukemia (AML) is a biologically heterogeneous hematologic malignancy characterized by clonal expansion of immature myeloid precursors, resulting in bone marrow failure and poor long-term survival [1,2]. Despite major advances in molecular profiling and supportive care, induction treatment for younger and medically fit patients has remained largely unchanged over recent decades. Conventional induction with cytarabine plus anthracycline (the “7+3” regimen) achieves complete remission (CR) rates of approximately 60–80% in younger adults overall. However, in patients with intermediate- and adverse-risk AML, expected composite complete remission rates with standard 7+3-based induction are closer to 50%, and relapse remains common despite initial response [3,4,5]. Moreover, treatment-related toxicities, prolonged cytopenias, infectious complications, and anthracycline-associated cardiac and organ toxicities remain important limitations of standard intensive induction.

The need for more effective and less toxic induction regimens is particularly relevant in patients with intermediate- and adverse-risk AML, in whom historical remission rates with standard chemotherapy remain suboptimal. Achieving deep remission and measurable residual disease (MRD) negativity is especially important in this population, as these patients are frequently candidates for allogeneic hematopoietic stem cell transplantation (allo-HSCT), where disease status at transplant strongly influences long-term outcomes [6,7].

Venetoclax, a selective BCL-2 inhibitor, enhances apoptosis in leukemic blasts and has demonstrated marked synergy with both hypomethylating agents and cytotoxic chemotherapy. In older or unfit patients, venetoclax-based combinations have significantly improved remission rates and overall survival, as demonstrated in the VIALE-A and VIALE-C studies [8,9]. More recently, venetoclax has been incorporated into intensive chemotherapy regimens for younger and fit patients with newly diagnosed AML [10,11,12].

Anthracycline-containing regimens, including 7+3 and FLAG-IDA-based approaches, remain established components of intensive frontline therapy for medically fit patients with AML. The clinical rationale for the present strategy was to explore whether a fludarabine/cytarabine backbone combined with venetoclax could provide effective cytoreduction while avoiding anthracycline exposure in selected transplant-eligible patients. Fludarabine/cytarabine combinations provide a potent antimetabolite backbone, while venetoclax has substantial pro-apoptotic synergy with intensive cytotoxic therapy [10,11]. Cumulative anthracycline exposure is associated with clinically relevant cardiac and other organ toxicities, particularly in older patients or those with cardiovascular comorbidity [13,14].

The G-CSF-free component of the regimen was based on a separate consideration. In the MRC AML-HR study, adding G-CSF to fludarabine and cytarabine did not improve outcomes in high-risk AML [15], and the AML15 experience showed that greater treatment intensity with FLAG-IDA reduced relapse but did not produce a clear overall-survival advantage over comparator intensive regimens [16]. Together with emerging evidence for venetoclax-containing intensive chemotherapy [10,11], these observations supported evaluation of a simplified FLA-VEN platform in routine practice.

FLT3 mutations are present in approximately 30% of AML cases and are associated with aggressive disease biology, increased relapse risk, and inferior survival [17]. Although the addition of midostaurin to standard 7+3 induction has improved outcomes in FLT3-mutated AML, relapse remains common even among patients who achieve initial remission [18]. Combining venetoclax with FLT3 inhibition is biologically attractive because preclinical studies suggest synergistic activity between BCL-2 inhibition and FLT3-targeted therapy, potentially resulting in deeper molecular responses and more durable remissions [19]. However, clinical experience with intensive FLA-VEN plus midostaurin combinations remains extremely limited, and there are very few published data evaluating this strategy in FLT3-mutated AML.

To date, real-world data evaluating anthracycline-free FLA-VEN-based induction in newly diagnosed AML remain limited, including in Türkiye, and clinical experience with FLA-VEN plus midostaurin in FLT3-mutated AML is particularly sparse. We therefore aimed to describe the response, safety, hematologic recovery, and transplant-bridging feasibility of this strategy in selected fit patients with intermediate- and adverse-risk AML, with post-transplant relapse and survival analyzed as secondary exploratory outcomes.

## 2. Materials and Methods

### 2.1. Study Design and Patient Selection

This retrospective, single-center study included all consecutive adult patients (≥18 years) with newly diagnosed acute myeloid leukemia (AML) treated at our institution between March 2024 and March 2026. AML diagnosis was established according to the 2022 World Health Organization (WHO) classification of myeloid neoplasms [20]. All patients were considered eligible for intensive induction chemotherapy at the time of diagnosis based on age, performance status, comorbidity profile, and organ function.

Patients with secondary AML, therapy-related AML, and AML with myelodysplasia-related changes were eligible. Patients with acute promyelocytic leukemia or a documented favorable-risk lesion were excludedFor this revision, ELN 2022 genetic risk was re-audited patient by patient using the retrievable baseline karyotype, FISH, FLT3-ITD, NPM1, and NGS results [21]. Broad baseline NGS was performed in 10 of 24 patients and was not performed in 14; conventional karyotyping was retrievable in 22 of 24. Therefore, an intermediate-risk designation was considered provisional when no favorable- or adverse-risk lesion was identified in the available tests, because untested ELN-relevant lesions could not be excluded, consistent with reported challenges in reliably applying the ELN 2022 genetic-risk classification when baseline molecular profiling is incomplete [22,23,24]. Six patients had a directly documented adverse-risk lesion. The complete de-identified evidence supporting each assignment is provided in Supplementary Table S1. No patient was excluded on the basis of TP53 status.

Patients were eligible if they had an Eastern Cooperative Oncology Group (ECOG) performance status of ≤2, creatinine clearance ≥ 30 mL/min, total bilirubin ≤ 1.5 × the upper limit of normal (ULN), and aspartate aminotransferase (AST) and alanine aminotransferase (ALT) ≤ 3 × ULN, unless liver function abnormalities were considered related to leukemic infiltration. Patients with prior venetoclax exposure, active central nervous system involvement, uncontrolled infection, New York Heart Association (NYHA) class III–IV heart failure, or left ventricular ejection fraction < 40% were excluded.

To reduce the risk of tumor lysis syndrome (TLS), baseline white blood cell counts were required to be ≤25 × 10^9^/L before treatment initiation. Cytoreductive therapy with hydroxyurea or low-dose cytarabine was allowed before induction according to physician discretion.

The study was approved by the local institutional ethics committee and conducted in accordance with the Declaration of Helsinki. Written informed consent for clinical treatment was obtained from all patients. Because this was a retrospective study, the requirement for separate study-specific informed consent for retrospective data collection and analysis was waived by the ethics committee.

### 2.2. Treatment Protocol

The FLA-VEN regimen consisted of fludarabine 30 mg/m^2^/day and cytarabine 1.5 g/m^2^/day administered intravenously on days 2–6. Anthracyclines and G-CSF priming were omitted. In FLT3-wild-type patients, venetoclax was initiated at 100 mg on day 1, 200 mg on day 2, and 400 mg on day 3, followed by 400 mg once daily through day 14.

In patients with FLT3-ITD, the same FLA backbone was used, but venetoclax was administered only on days 1–7 and was followed sequentially by midostaurin 50 mg orally twice daily on days 8–21. The shorter venetoclax exposure was a pragmatic institutional modification intended to limit prolonged overlap of intensive chemotherapy with two targeted agents, thereby reducing overlapping myelosuppression and drug-interaction complexity. This sequential schedule was not derived from comparative evidence and should not be interpreted as an established optimal duration.

Following allo-HSCT, all FLT3-ITD-positive patients received sorafenib maintenance therapy for a planned duration of two years. In Türkiye, access to other FLT3 inhibitors for post-transplant maintenance remained limited during the study period because agents such as quizartinib and gilteritinib were not reimbursed for this indication.

The induction schedules are summarized in Table 1.

Post-remission therapy was planned with the intention of proceeding to allo-HSCT in CR1 for transplant-eligible patients with intermediate- or adverse-risk AML and an available donor. After achievement of CR, patients were referred for transplantation as soon as donor identification, transplant work-up, and logistical conditions permitted. There was no protocol-mandated fixed number of consolidation cycles. Selected patients could receive bridging high-dose cytarabine while awaiting allo-HSCT according to physician discretion and logistical timelines. Because the number and timing of pre-transplant consolidation cycles were not uniformly captured, their association with subsequent post-transplant outcomes could not be formally analyzed.

### 2.3. Supportive Care

All patients received standardized antimicrobial prophylaxis, including antiviral prophylaxis with acyclovir or valacyclovir, antibacterial prophylaxis with levofloxacin, and antifungal prophylaxis. Because of potential CYP3A4-mediated drug–drug interactions, azole antifungals were avoided during venetoclax administration whenever possible, and micafungin (100 mg/day intravenously) was preferred. If azole antifungals were required because of suspected or proven invasive fungal infection, venetoclax doses were reduced according to institutional protocols.

TLS prophylaxis consisted of intravenous hydration and allopurinol or rasburicase, initiated one day before venetoclax ramp-up. Laboratory monitoring for TLS was performed daily during treatment initiation and subsequently as clinically indicated.

Febrile neutropenia was managed according to institutional protocols using empiric broad-spectrum intravenous antibiotics. In patients with persistent fever, prolonged neutropenia, or clinical/radiological suspicion of invasive fungal infection, antifungal therapy was initiated and managed according to current ECIL recommendations [25]. Red blood cell transfusions were administered according to a restrictive transfusion strategy, generally when hemoglobin levels fell below 7–8 g/dL or in the presence of symptomatic anemia. Platelet transfusions were administered prophylactically when platelet counts were below 10 × 10^9^/L in clinically stable afebrile patients and below 20 × 10^9^/L in patients with fever or additional bleeding risk factors, in accordance with current transfusion guidelines [26,27]. Growth factor support with G-CSF was not routinely administered during induction or until hematologic recovery, as previous studies have shown that although G-CSF may shorten the duration of neutropenia and hospitalization, it does not appear to improve remission rates, relapse risk, or overall survival in AML patients receiving induction chemotherapy [28].

### 2.4. Molecular Analysis

FLT3 mutation status was assessed using PCR-based fragment analysis targeting FLT3 internal tandem duplication (ITD) and tyrosine kinase domain (TKD) mutations at codons D835 and I836. Molecular testing was performed at the GENETİKS Genetic Diseases Evaluation Center (İzmir, Türkiye). Genomic DNA was extracted from bone marrow aspirates followed by fluorescent PCR amplification and capillary electrophoresis; the analytic sensitivity was approximately 5% mutant allele burden. Samples with no detectable ITD or TKD mutations were classified as FLT3 wild type. The revised ELN 2022 assignment used only retrievable patient-level findings. FLT3-ITD without a documented adverse-risk lesion was classified as intermediate risk irrespective of allelic ratio. TP53, SRSF2, and SF3B1 mutations and reported t(9;22)/BCR: ABL1 positivity supported adverse-risk assignment. One patient had an IDH1 mutation, which is not independently classified as favorable or adverse in the ELN 2022 genetic-risk system; this patient was therefore designated provisional intermediate risk in the absence of another documented ELN-defining lesion. Patients without an identified favorable- or adverse-risk lesion were designated provisional intermediate risk, explicitly acknowledging possible under-classification from incomplete baseline profiling.

### 2.5. Endpoints and Response Assessment

The primary endpoints were complete remission (CR) rate after the first induction cycle and treatment safety. Bone marrow response assessment was performed in all patients approximately 21–28 days after induction according to the 2022 ELN response criteria [21]. Response assessments were performed locally by hematopathologists at our institution. Only complete remission (CR) was recorded in this cohort; no patients met the criteria for complete remission with incomplete hematologic recovery (CRi). All patients received one protocol-defined induction cycle before the primary response assessment, and no additional FLA-VEN induction cycle was administered before determination of the primary response endpoint.

Standardized measurable residual disease (MRD) assessment was not routinely available for the entire cohort. In particular, multiparameter flow-cytometric MRD was not systematically performed in FLT3-wild-type patients because of the retrospective real-world setting and limited routine availability. In patients with a detectable FLT3-ITD clone at diagnosis, the same PCR-based fragment assay was repeated when feasible as an available molecular response marker. Because the assay sensitivity was approximately 5%, these results do not meet contemporary sensitivity thresholds for formal MRD assessment; therefore, throughout the revised manuscript we report FLT3-ITD as ‘undetectable by the available PCR fragment assay’ rather than ‘MRD-negative.’

Adverse events were graded according to the Common Terminology Criteria for Adverse Events (CTCAE), version 5.0 [29]. Secondary endpoints included time to neutrophil recovery (>0.5 × 10^9^/L), time to platelet recovery (>50 × 10^9^/L without transfusion support), duration of hospitalization, rate of allo-HSCT, relapse incidence, transplant-related mortality (TRM), and overall mortality. Infectious outcomes were reviewed retrospectively from the available clinical records. Invasive fungal infection and infection-related mortality were specifically captured; however, microbiologically documented bacteremia was not maintained as a separate prespecified variable in the source analysis dataset, precluding a reliable cohort-level bacteremia incidence from the currently available data.

### 2.6. Statistical Analysis

Descriptive statistics were used to summarize baseline characteristics, treatment responses, and adverse events. Categorical variables were reported as frequencies and percentages, whereas continuous variables were presented as medians and ranges. Because of the limited sample size and retrospective design, no formal hypothesis testing was planned.

Overall survival (OS) was defined as the time from diagnosis to death from any cause or last follow-up, with a database cutoff of 30 August 2026. Transplant-related mortality (TRM) was defined as any death occurring after allo-HSCT in the absence of preceding leukemia relapse, irrespective of whether death occurred before or after day 30, day 60, or day 100. Thus, TRM was not restricted to a fixed early post-transplant landmark and was assessed over the entire post-transplant follow-up period. The separately reported 60-day mortality endpoint referred to all-cause mortality within 60 days after initiation of induction therapy.

Overall survival was estimated using the Kaplan–Meier method from patient-level time-to-event and censoring data. Median follow-up was estimated using the reverse Kaplan–Meier method. Pointwise 95% confidence intervals for landmark OS estimates were calculated using Greenwood variance with log–log transformation. Relapse events were summarized descriptively from the time of complete remission; no time-to-relapse or competing-risk cumulative-incidence estimate was calculated. Statistical analyses were conducted using IBM SPSS Statistics version 26 (IBM Corp., Armonk, NY, USA).

## 3. Results

### 3.1. Baseline Characteristics

A total of 24 consecutive adults with newly diagnosed AML were included between March 2024 and March 2026. Baseline demographic and clinical characteristics are summarized in Table 2. The median age was 49 years (range, 22–64), 13 patients (54.2%) were female, 4 (16.7%) were aged ≥60 years, and the median ECOG performance status was 0 (range, 0–1).

On patient-level re-audit, 18 patients (75.0%) were designated provisional ELN 2022 intermediate risk and 6 (25.0%) had a directly documented adverse-risk lesion. FLT3-ITD was detected in 8 patients (33.3%), whereas 16 (66.7%) were FLT3 wild type. Conventional karyotyping was retrievable in 22 of 24 patients, FISH results in all 24, and broad NGS results in 10 of 24; broad NGS was not performed in 14 patients. Supplementary Table S1 reports the de-identified findings and the rationale for every assignment.

### 3.2. Induction Response and Safety

After the protocol-defined first induction cycle, 21 of 24 patients (87.5%) achieved complete remission (Table 3); no patient met criteria for CRi. A response breakdown by FLT3 status is reported in Section 3.3; a breakdown by ELN 2022 risk category is not reported separately, because the patient-level re-audit could not be reliably linked to response data at the individual-patient level (see Limitations). Three patients (12.5%) had primary refractory disease.

Induction safety was favorable in this selected cohort: 60-day all-cause mortality was 0/24 (0%), no patient required intensive care unit admission, and no grade 3–4 non-hematologic toxicity, including severe mucositis or organ toxicity, was documented.

Venetoclax reached the protocol-defined target dose in all patients without permanent treatment discontinuation. Micafungin was used for antifungal prophylaxis to minimize CYP3A4-mediated interactions. Febrile neutropenia was managed according to institutional protocols; no invasive fungal infection or infection-related death occurred. Microbiologically documented bacteremia was not separately enumerated in the source dataset and therefore could not be reported reliably.

Among patients achieving CR, median neutrophil recovery was 17 days (range, 11–30), median platelet recovery was 21 days (range, 13–29), and median hospitalization duration was 28 days (range, 16–40).

### 3.3. FLT3 Subgroup and Molecular Response

Among FLT3-wild-type patients treated with FLA-VEN, 14 of 16 (87.5%) achieved CR. In the FLT3-ITD subgroup treated with FLA-VEN plus midostaurin, 7 of 8 (87.5%) achieved CR. FLT3-ITD was undetectable by the available PCR fragment assay in all seven responders before allo-HSCT. Because the assay sensitivity was approximately 5%, this finding is reported as molecular undetectability by the available test and not as formal MRD negativity.

### 3.4. Post-Remission Therapy and Allo-HSCT

All 21 patients who achieved CR proceeded to allo-HSCT in first remission (87.5% of the total cohort). Transplantation was performed as soon as donor identification, work-up, and logistics permitted. There was no uniform protocol-mandated consolidation schedule; selected patients received bridging HD-AraC while awaiting transplantation. The exact number and timing of consolidation cycles were not uniformly captured and were therefore not analyzed in relation to subsequent transplant outcomes.

### 3.5. Post-Transplant Relapse and Long-Term Survival

The database cutoff for survival follow-up was 30 August 2026, and median follow-up by reverse Kaplan–Meier was 20.9 months. The three patients with primary refractory disease died of refractory AML within the first year. No relapse occurred during the first year. The Kaplan–Meier estimated 12-month OS for the entire cohort was 83.3% (95% CI, 61.5–93.4%).

After the first year, one patient relapsed and subsequently died, and four additional patients died after allo-HSCT without preceding relapse and were classified as transplant-related mortality (TRM). TRM therefore occurred in 4 of 21 transplanted patients (19.0%; 16.7% of the entire cohort). At last follow-up, eight deaths had occurred: three from refractory AML, one after relapse, and four from TRM. The estimated 24-month OS was 54.4% (95% CI, 25.5–76.3%), with only five patients remaining at risk at 24 months; median OS was not reached. The Kaplan–Meier overall survival curve is shown in Figure 1.

No relapse occurred during the first year after complete remission. During follow-up, one relapse was observed among the 21 patients who achieved complete remission and underwent allo-HSCT (1/21, 4.8%); this is an observed proportion, not a 2-year cumulative-incidence estimate. Because all responders proceeded to allo-HSCT, relapse, TRM, and longer-term survival reflect the combined effects of induction, transplantation, and post-transplant management and are presented as secondary exploratory outcomes rather than outcomes attributable solely to the induction regimen.

## 4. Discussion

In this single-center retrospective study, we report real-world outcomes of anthracycline-free and G-CSF-free FLA-VEN, with the addition of midostaurin in FLT3-mutated cases, as frontline induction therapy in newly diagnosed intermediate- and adverse-risk AML. The main findings of our analysis include a high complete remission (CR) rate, rapid hematologic recovery, absence of early mortality, and a high rate of successful bridging to allogeneic hematopoietic stem cell transplantation (allo-HSCT).

The CR rate of 87.5% was numerically high and within the range reported with recent venetoclax-containing intensive regimens [10,11]. Because no concurrent comparator was included, historical 7+3- or FLAG-based results are used only as clinical context rather than as a formal efficacy comparison.

Anthracycline omission was intended as a toxicity-sparing strategy. Anthracycline-containing intensive chemotherapy remains a standard frontline approach, but cumulative cardiac and organ toxicity remains clinically relevant, particularly in older patients and those with cardiovascular comorbidity [14]. The absence of 60-day mortality in this selected cohort is therefore encouraging but requires confirmation in comparative studies.

G-CSF priming was omitted because randomized AML studies have not consistently shown improvements in remission, relapse, or overall survival with its incorporation into induction [15]. The present experience therefore evaluates a deliberately simplified fludarabine/cytarabine/venetoclax backbone.

Hematologic recovery in our cohort was relatively prompt, with median neutrophil and platelet recovery times of 17 and 21 days, respectively. No grade 3–4 non-hematologic toxicities were documented, and no patients required intensive care unit admission. Venetoclax was administered to the protocol-defined target dose in all patients without permanent treatment discontinuation, largely because azole antifungals were avoided and micafungin was used for antifungal prophylaxis. Importantly, no invasive fungal infections or infection-related deaths were observed. These findings suggest that FLA-VEN is feasible and well tolerated in a carefully selected population of fit, transplant-eligible patients.

The FLT3-mutated subgroup is of particular interest. FLA-VEN plus sequential midostaurin produced CR in 7 of 8 patients, and FLT3-ITD became undetectable by the available PCR fragment assay in all seven responders before transplantation. Because the assay sensitivity was approximately 5%, this does not represent formal MRD negativity. The sequential venetoclax/midostaurin schedule was chosen pragmatically to limit overlapping myelosuppression and treatment-interaction complexity, and its optimal duration remains undefined. These observations warrant prospective evaluation of intensive venetoclax plus FLT3-inhibitor strategies. These observations are biologically consistent with preclinical data demonstrating synergistic antileukemic activity when BCL-2 inhibition is combined with FLT3 inhibitors [19,30]. Clinical data with azacitidine, venetoclax, and gilteritinib further support the activity of combined BCL-2 and FLT3 targeting, although that lower-intensity treatment context differs from the intensive platform evaluated here [31].

The high rate of allo-HSCT in our study reflects both institutional strategy and the high proportion of patients achieving remission after induction therapy. All patients who achieved CR proceeded to allo-HSCT in first remission, resulting in a transplantation rate of 87.5%. This finding was facilitated by donor availability through national registry systems and reflects the institutional preference to offer transplantation to all fit patients with intermediate- or adverse-risk AML in first remission.

The survival pattern is best interpreted by separating induction outcomes from subsequent transplant-related outcomes. The induction phase was characterized by an 87.5% CR rate and 0% 60-day mortality. Thereafter, every responder underwent allo-HSCT, making later relapse, TRM, and OS composite consequences of induction efficacy, transplant selection, conditioning, and post-transplant care. The 24-month OS estimate of 54.4% was based on only five patients at risk and had a wide 95% CI (25.5–76.3%). Later mortality was driven predominantly by non-relapse post-transplant events, with four TRM deaths compared with one relapse-related death.

Several limitations of this study should be acknowledged. First, the retrospective design, single-center setting, and relatively small sample size limit the generalizability of our findings. Second, the absence of a contemporaneous control group precludes direct comparison with standard 7+3-based induction. We considered the potential value of an institutional historical 7+3 comparator; however, a sufficiently comparable cohort with the same eligibility criteria, ELN 2022 risk assignment, molecular testing availability, transplant strategy, and supportive-care era was not available within the present study dataset. A post hoc unmatched historical comparison would therefore be highly vulnerable to selection, temporal, and treatment-allocation bias and could be misleading. Published historical outcomes are consequently discussed only as contextual benchmarks. Third, the study population was highly selected, consisting predominantly of younger, fit, transplant-eligible patients with ECOG performance status 0–1, which may have contributed to the favorable early outcomes observed.

In addition, comprehensive baseline NGS was not routinely performed, and 18 intermediate-risk assignments were therefore provisional because untested adverse-risk lesions could not be excluded. The patient-level re-audit identified six cases with directly documented adverse-risk abnormalities and corrected the previously reported aggregate distribution. The revised classification and all retrievable karyotype, FISH, and molecular results are provided in Supplementary Table S1. The risk-stratified CR comparison was removed because the reclassified patient-level response data were not available in a form that permitted a reliable recalculation. Standardized multiparameter flow-cytometric MRD was also not routinely available in FLT3-wild-type patients. In FLT3-ITD cases, the PCR fragment assay was used only as an available molecular response marker; its approximately 5% sensitivity is insufficient for formal MRD designation. The FLT3-mutated subgroup was small, and findings with FLA-VEN plus midostaurin should be considered hypothesis-generating rather than definitive. Furthermore, adverse events may have been underreported because of the retrospective nature of the study, particularly for lower-grade toxicities. Infectious toxicity ascertainment was also retrospective; although invasive fungal infection and infection-related mortality were captured, bacteremia was not systematically enumerated as a separate variable in the source analysis dataset.

Finally, the high transplantation rate and the incidence of transplant-related mortality reflect institutional practice patterns and the transplant-eligible nature of the cohort. Post-remission management before allo-HSCT was not uniform; selected patients could receive bridging HD-AraC, and the exact number and timing of consolidation cycles were not uniformly captured. This heterogeneity prevents a reliable assessment of whether pre-transplant consolidation influenced TRM. Longer follow-up and prospective multicenter validation with standardized molecular profiling, MRD assessment, and post-remission treatment are required.

Despite these limitations, the study provides real-world feasibility data for anthracycline-free and G-CSF-free FLA-VEN, with sequential midostaurin in FLT3-ITD AML, in selected fit patients with intermediate- and adverse-risk disease. The high first-cycle CR rate, absence of early mortality, and successful transition of all responders to allo-HSCT support prospective multicenter evaluation with standardized molecular profiling and a contemporaneous anthracycline-containing comparator.

## 5. Conclusions

Anthracycline-free and G-CSF-free FLA-VEN, with sequential midostaurin in FLT3-ITD cases, was feasible in this selected single-center cohort and was associated with an 87.5% first-cycle CR rate, no 60-day mortality, and successful transition of all responders to allo-HSCT.

Prospective multicenter studies with standardized molecular profiling, MRD assessment, and an appropriate concurrent anthracycline-containing comparator are needed to define the comparative efficacy, optimal venetoclax duration, and long-term clinical value of this strategy.

## Figures and Tables

**Figure 1:**
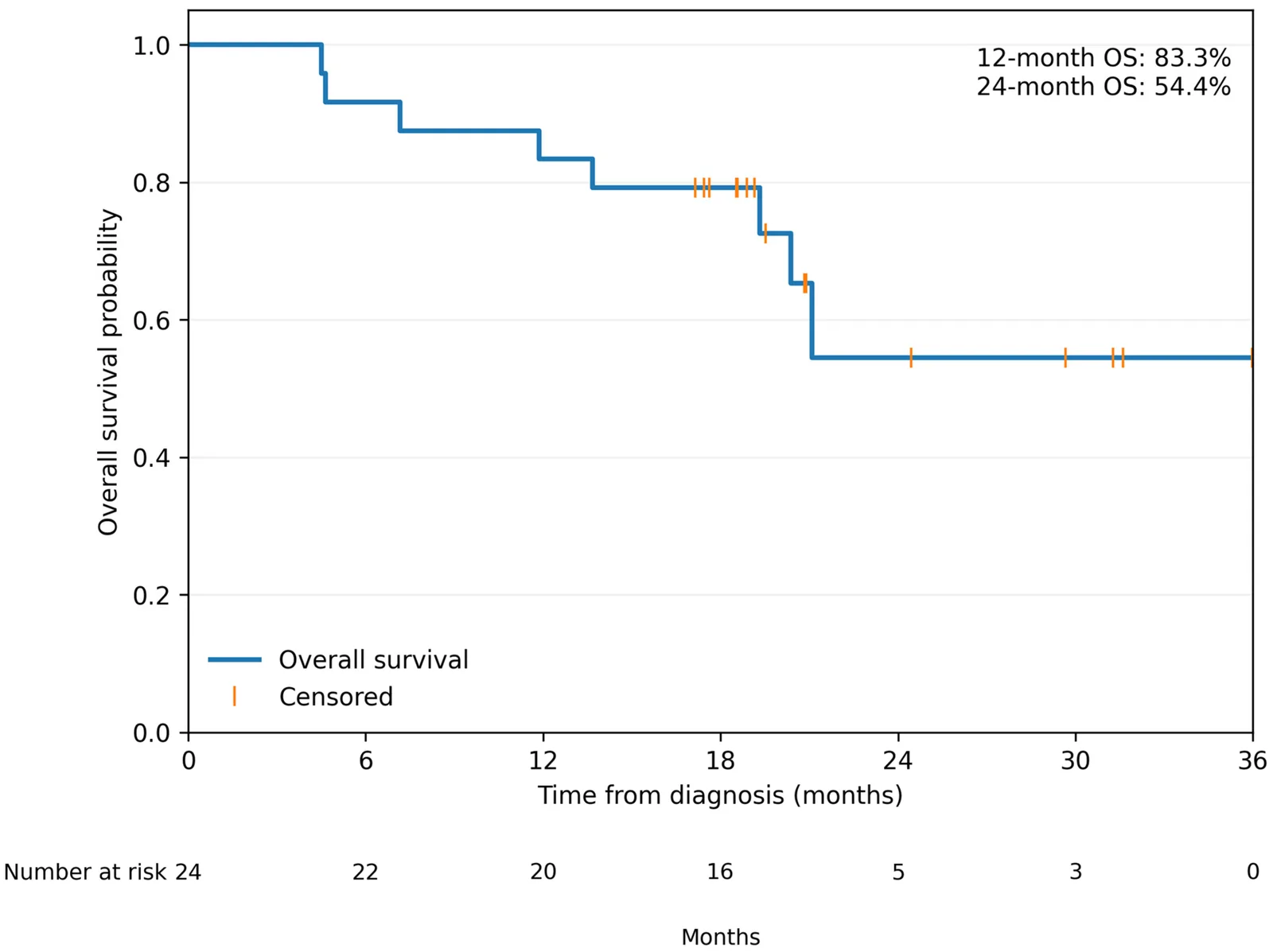
Kaplan–Meier overall survival curve for the entire study population (*N* = 24). Overall survival was measured from diagnosis to death from any cause or last follow-up (database cutoff, 30 August 2026). Tick marks denote censored observations; numbers at risk are shown below the x-axis. The estimated OS was 83.3% at 12 months and 54.4% at 24 months; only five patients remained at risk at 24 months, and median OS was not reached.

**Table 1:** Induction treatment protocol.

FLA-VEN + Midostaurin (FLT3-ITD)	FLA-VEN	Component
30 mg/m^2^/day IV, days 2–6	30 mg/m^2^/day IV, days 2–6	Fludarabine
1.5 g/m^2^/day IV, days 2–6	1.5 g/m^2^/day IV, days 2–6	Cytarabine
100 mg day 1; 200 mg day 2; 400 mg/day days 3–7	100 mg day 1; 200 mg day 2; 400 mg/day days 3–14	Venetoclax
50 mg orally twice daily, days 8–21	Not administered	Midostaurin
Not administered	Not administered	Anthracycline
Not administered	Not administered	G-CSF priming

Note: In FLT3-ITD-positive patients, venetoclax and midostaurin were administered sequentially. The 7-day venetoclax duration was a pragmatic institutional strategy to limit prolonged overlapping myelosuppression and drug-interaction complexity; it was not based on a comparative dose-duration study.

**Table 2:** Baseline Demographic, Clinical, and Available Molecular Characteristics.

Characteristic	Value
Number of patients	24
Median age, years (range)	49 (22–64)
Age ≥ 60 years, *n* (%)	4 (16.7%)
Female sex, *n* (%)	13 (54.2%)
Median ECOG performance status (range)	0 (0–1)
Provisional ELN 2022 intermediate risk, *n* (%)	18 (75.0%)
ELN 2022 adverse risk supported by a documented lesion, *n* (%)	6 (25.0%)
FLT3-ITD mutated, *n* (%)	8 (33.3%)
FLT3 wild type, *n* (%)	16 (66.7%)
Comprehensive baseline NGS	Broad baseline NGS available in 10/24; not performed in 14/24
TP53 mutation status	Available when tested; TP53 mutation documented in 3/24
Standardized flow-cytometric MRD	Not routinely available
Other ELN-relevant gene mutations	Patient-level available findings reported in Supplementary Table S1
Conventional cytogenetic/FISH information	Karyotype retrievable in 22/24; FISH retrievable in 24/24

**Table 3:** Treatment Outcomes and Safety.

Outcome	Value
Complete remission (CR), *n* (%)	21/24 (87.5%)
CR in FLT3 wild-type AML	14/16 (87.5%)
CR in FLT3-ITD mutated AML	7/8 (87.5%)
FLT3-ITD undetectable before allo-HSCT by available PCR fragment assay	7/7 (100%)
Proceeding to allo-HSCT in first remission	21/24 (87.5%)
60-day mortality	0/24 (0%)
Median neutrophil recovery, days (range)	17 (11–30)
Median platelet recovery, days (range)	21 (13–29)
Median hospitalization duration, days (range)	28 (16–40)
Primary refractory disease, *n* (%)	3/24 (12.5%)
Estimated 12-month overall survival	83.3% (95% CI, 61.5–93.4%)
Estimated 24-month overall survival	54.4% (95% CI, 25.5–76.3%)
Relapse within the first year after CR	0/21 (0%)
Observed relapse after CR during follow-up	1/21 (4.8%)
TRM after allo-HSCT	4/21 (19.0%); 4/24 (16.7%) overall
Microbiologically documented bacteremia	Not systematically enumerated in source dataset

## Data Availability

All data generated or analyzed during this study are included in this article. Further enquiries can be directed to the corresponding author.

## Supplementary Materials

The following supporting information can be downloaded at: https://www.mdpi.com/article/10.3390/cancers18193132/s1, Table S1. Patient-Level Genetic Findings and ELN 2022 Assignment.
