# Supplementary material for: Anthracycline-Free FLA-VEN with or Without Midostaurin in Newly Diagnosed Intermediate- and Adverse-Risk AML: A Single-Center Retrospective Study
Source: Cancers (Basel). 2026 Sep 27;18(19):3132. doi: 10.3390/cancers18193132 (PMC13664420; doi:10.3390/cancers18193132)
Supplement: Supplementary file 1 [file cancers-18-03132-s001.zip › cancers-4541468-supplementary.pdf]

Table S1. Patient Level Genetic Findings and ELN 2022 Assignment. The table reports all retrievable baseline findings for the 24-patient manuscript cohort. Study identifiers are de-identified. "Provisional intermediate" indicates that no favorable- or adverse-risk lesion was identified in the available tests, but incomplete baseline profiling prevents exclusion of an untested adverse-risk lesion.

ID	Karyotype	FISH	NGS or molecular panel	FLT3-ITD	NPM1	Revised ELN 2022 assignment and basis
P01	Normal	Negative	Not performed	Positive	Negative	Provisional intermediate: FLT3-ITD without a documented adverse-risk lesion; incomplete baseline profiling may under-classify risk
P02	Normal	Negative	Not performed	Positive	Negative	Provisional intermediate: FLT3-ITD without a documented adverse-risk lesion; incomplete baseline profiling may under-classify risk
P03	Normal	Negative	Not performed	Negative	Negative	Provisional intermediate: No favorable- or adverse-risk lesion identified in the available results; incomplete baseline profiling may under-classify risk
P04	Normal	Negative	SRSF2; TET2	Negative	Negative	Adverse: SRSF2 mutation (myelodysplasia-related gene mutation)
P05	Normal	Negative	Not performed	Negative	Negative	Provisional intermediate: No favorable- or adverse-risk lesion identified in the available results; incomplete baseline profiling may under-classify risk
P06	Normal	Negative	Not performed	Negative	Negative	Provisional intermediate: No favorable- or adverse-risk lesion identified in the available results; incomplete baseline profiling may under-classify risk
P07	Normal	Negative	TP53 positive	Negative	Negative	Adverse: TP53 mutation
P08	Normal	Negative	Not performed	Negative	Negative	Provisional intermediate: No favorable- or adverse-risk lesion identified in the available results; incomplete baseline profiling may under-classify risk
P09	Normal	Negative	Not performed	Negative	Negative	Provisional intermediate: No favorable- or adverse-risk lesion identified in the available results; incomplete baseline profiling may under-classify risk
P10	Normal	Negative	GATA2; NRAS; SF3B1	Negative	Negative	Adverse: SF3B1 mutation (myelodysplasia-related gene mutation)
P11	Normal	Negative	Not performed	Negative	Negative	Provisional intermediate: No favorable- or adverse-risk lesion identified in the available results; incomplete baseline profiling may under-classify risk

ID	Karyotype	FISH	NGS or molecular panel	FLT3-ITD	NPM1	Revised ELN 2022 assignment and basis
P12	Normal	**Negative**	FLT3; WT1	Positive	Negative	Provisional intermediate: FLT3-ITD without a documented adverse-risk lesion; incomplete baseline profiling may under-classify risk
P13	Not performed	Negative	TP53 positive	Negative	Negative	Adverse: TP53 mutation
P14	Normal	Negative	Not performed	Positive	Negative	Provisional intermediate: FLT3-ITD without a documented adverse-risk lesion; incomplete baseline profiling may under-classify risk
P15	Normal	Negative	DNMT3A; NPM1	Positive	Positive	Provisional intermediate: FLT3-ITD without a documented adverse-risk lesion; incomplete baseline profiling may under-classify risk
P16	Normal	Negative	Not performed	Positive	Negative	Provisional intermediate: FLT3-ITD without a documented adverse-risk lesion; incomplete baseline profiling may under-classify risk
P17	Normal	Negative	Not performed	Negative	Negative	Provisional intermediate: No favorable- or adverse-risk lesion identified in the available results; incomplete baseline profiling may under-classify risk
P18	Normal	Negative	Not performed	Negative	Negative	Provisional intermediate: No favorable- or adverse-risk lesion identified in the available results; incomplete baseline profiling may under-classify risk
P19	Normal	5q abnormality reported	TP53 positive	Negative	Negative	Adverse: TP53 mutation; abnormal 5q FISH result
P20	Normal	Negative	Not performed	Positive	Negative	Provisional intermediate: FLT3-ITD without a documented adverse-risk lesion; incomplete baseline profiling may under-classify risk
P21	Normal	Negative	Not performed	Positive	Negative	Provisional intermediate: FLT3-ITD without a documented adverse-risk lesion; incomplete baseline profiling may under-classify risk
P22	Normal	Negative	IDH1 mutation detected	Negative	Negative	Provisional intermediate: IDH1 mutation is not independently favorable or adverse in ELN 2022; no other documented ELN-defining lesion was identified, and incomplete profiling may under-classify risk
P23	Not available	t(9;22) reported positive	t(9;22) reported positive	Negative	Negative	Adverse: Reported t(9;22)/BCR::ABL1 positivity

ID	Karyotype	FISH	NGS or molecular panel	FLT3-ITD	NPM1	Revised ELN 2022 assignment and basis
P24	Normal	Negative	Negative	Negative	Negative	Provisional intermediate: No favorable- or adverse-risk lesion identified in the available results; incomplete baseline profiling may under-classify risk

Abbreviations: ELN, European LeukemiaNet; FISH, fluorescence in situ hybridization; NGS, next-generation sequencing; ITD, internal tandem duplication. A negative FISH result denotes no abnormality detected on the panel performed; it does not establish that every ELN-defining rearrangement was tested. "NGS or molecular panel" reproduces the retrievable reported result; panel composition and coverage were not uniformly available. Clarification: P22 had an IDH1 mutation only. IDH1 is not independently classified as favorable or adverse in the ELN 2022 genetic-risk system.
